# A training program for improving the capacity of infection high-throughput sequencing and diagnosis in China

**DOI:** 10.1186/s12909-024-05100-2

**Published:** 2024-02-14

**Authors:** Dong Zhang, Yunfeng Cheng, Yuan Ji, Qing Miao, Bojiang Chen, Jing Wang, Guoqiu Wu, Chenyan Yuan, Guangjuan Zheng, Han Liu, Xinmin Qiu, Jie Gong, Hongping Ba, Liping Pan, Xiaoling Ma, Yingjie Qi, Yuru Shi, Qi Zhang, Dan Li, Yingchun Xu

**Affiliations:** 1 Department of Clinical Laboratory, State Key Laboratory of Complex Severe and Rare Diseases, Peking Union Medical College Hospital, Chinese Academy of Medical Sciences and Peking Union Medical College, Beijing, People’s Republic of China; 2 Key Laboratory of Pathogen Infection Prevention and Control (Peking Union Medical College), Ministry of Education, Beijing, China; 3 Zhongshan Hospital, Fudan University, 180 Fenglin Rd, 200032 Shanghai, China; 4 Precision Medicine Center, Precision Medicine Key Laboratory of Sichuan Province, West China Hospital, Sichuan University, 610041 Chengdu, China; 5 Center of Clinical Laboratory Medicine, Zhongda Hospital, Southeast University, 210009 Nanjing, Jiangsu China; 6 Genetic Testing Lab, The Second Clinical College, Guangzhou University of Chinese Medicine, Guangzhou, China; 7 Department of Clinical Laboratory, Wuhan Center for Clinical Laboratory, Wuhan, Hubei China; 8 Department of Laboratory Medicine, Division of Life Sciences and Medicine, The First Affiliated Hospital of USTC, University of Science and Technology of China, 230000 Hefei, Anhui China; 9 Beijing life oasis public service center, Beijing, China; 10 BGI Genomics, 518083 Shenzhen, China

**Keywords:** Pathogen, Metagenomic, Diagnosis, Localization, Small-scale training course

## Abstract

**Background:**

Infectious diseases are a serious threat to human especially since the COVID-19 outbreak has proved the importance and urgency of their diagnosis and treatment again. Metagenomic next-generation sequencing (mNGS) has been widely used and recognized in clinical and carried out localized testing in hospitals. Increasing the training of mNGS detection technicians can enhance their professional quality and more effectively realize the application value of the hospital platform.

**Methods:**

Based on the initial theoretical understanding and practice of the mNGS platform for localization construction, we have designed a training program to enhance the ability of technicians to detect pathogens by utilizing mNGS, and hence to conduct training practices nationwide.

**Results:**

Until August 30, 2022, the page views of online classes have reached 51,500 times and 6 of offline small-scale training courses have been conducted. A total of 67 trainees from 67 hospitals have participated in the training with a qualified rate of 100%. After the training course, the localization platform of 1 participating hospital has been put into use, 2 have added the mNGS localization platform for admission, among which 3 have expressed strong intention of localization.

**Conclusions:**

This study focuses on the training procedures and practical experience of the project which is the first systematic standardized program of mNGS in the world. It solves the training difficulties in the current industry, and effectively promotes the localization construction and application of mNGS in hospitals. It has great development potential in the future and is worth further promotion.

## Background

Infectious diseases with excessive morbidity and mortality are a constant concern for human health and cause different clinical symptoms [1].Timely identification of the pathogen can effectively improve the prognosis of infected patients [2–4]. mNGS directly sequenced all microorganisms and host nucleic acids in the sample for unbiased sequencing. Combined with pathogen database and specific algorithms, mNGS can detect the sequence of pathogenic microorganisms that may be contained in the sample. Recently, mNGS has moved from scientific research into clinical practice and is also changing the pattern of disease diagnosis and treatment [5–7]. The localized mNGS detection platform enables accurate diagnosis of early-stage infectious agents, monitoring of targeted resistance, and tracking of hospital infections by specific agents with high coverage, thus facilitating rational standardized use and control of antibiotics.

China consensus first mentioned the next-generation sequencing (NGS) may be used in clinical applications in the future in 2017 [8]. “2018 Chinese guidelines for the diagnosis and treatment of adults eith hospital-acquired and ventilator associated pneumonia” introduced the advantages of metagenomics high-throughput sequencing in clinical infection detection and diagnosis for the first time [9]. In recent years, more than 10 expert consensus reports related to mNGS have been published in China, covering areas such as the central nervous system, respiratory system, and pediatric infections, but its clinical applications are still to be standardized [10–14]. Currently, a growing number of medical institutions are conducting or preparing to conduct mNGS testing. Clinical applications of mNGS suffer from a number of problems, such as relatively high technical difficulty, relatively complex operational process, relatively difficult reporting interpretation, clinical application specifications that need to be improved, unsatisfactory results of inter-laboratory quality assessment, and lack of a unified and standardized training mechanism in the industry. In light of the current situation, it is urgent to train the mNGS for local construction in the industry and set up a standardized training mechanism. There is an urgent need to raise the standard of technological applications.

Combined with the training context, we effectively address the related issues faced by the localization detection criteria of mNGS through the precise setting of the training content, the scientific design of the training mode, and, from a practical point of view, accurate teaching. Through the sharing of localization experience among top medical institutions, we can help popularize the localization of mNGS detection technology in grassroots hospitals, promote multidisciplinary interactions between laboratories and clinical departments, and improve the comprehensive diagnosis and treatment capacity of infectious diseases in hospitals. Meanwhile, the current situation of relevant personnel training in primary medical institutions should be improved to help improve the technical level and qualification of mNGS related detection personnel in primary hospitals, better and faster understand and use the detection technology, more effectively realize the application value of the localization of mNGS detection technology, and promote the standardization construction of localized clinical detection and application of mNGS.

**Fig. 1:**
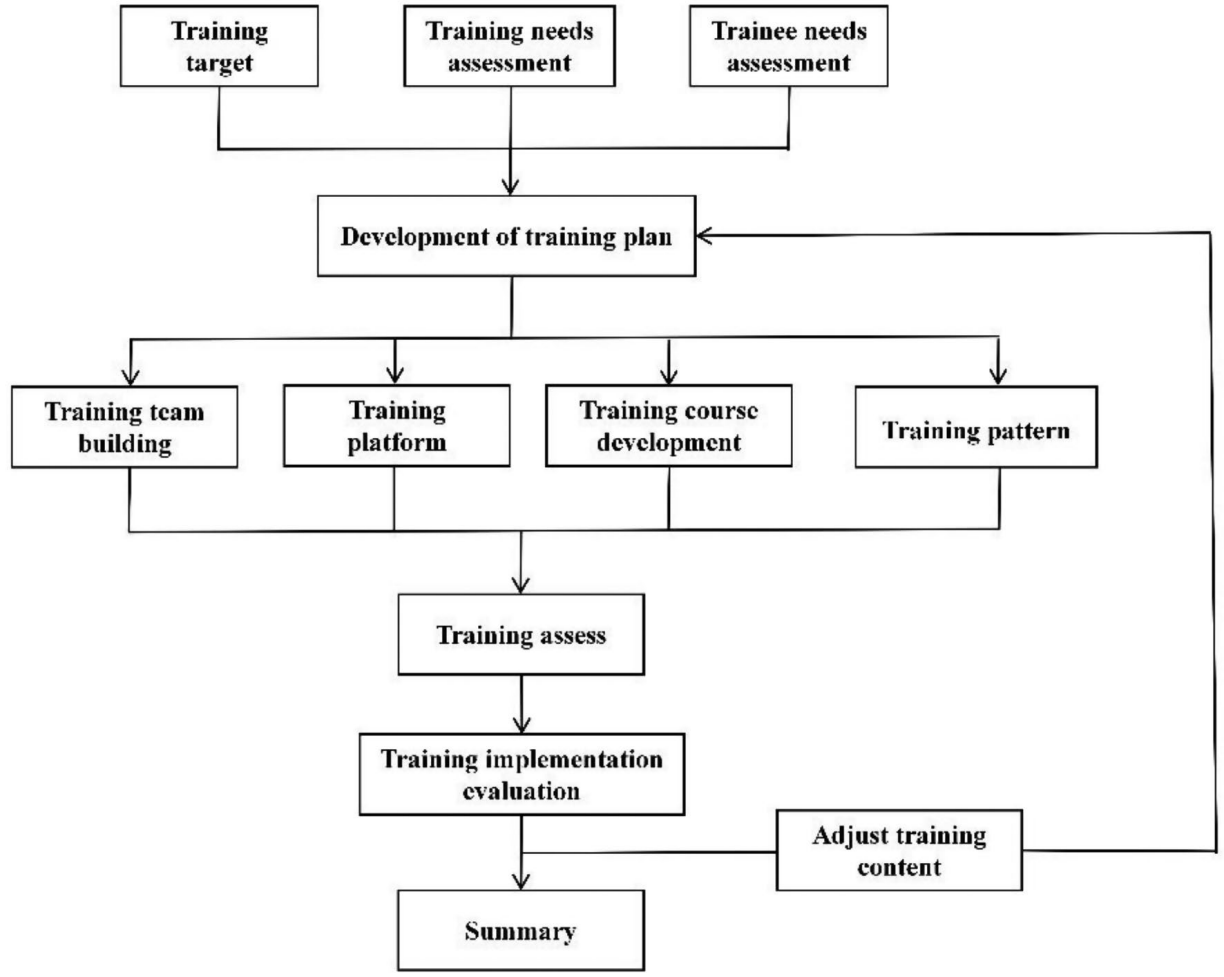
Flow chart of the training program

Based on the above background, we have established a relatively complete talent training program and plan. The main implementation process of the whole training project is shown in Fig. 1. We hope that through this training program, the technical level of mNGS testing personnel including teaching hospitals and participating hospitals can be improved, the application value of the hospital platform can be more effectively realized, and the testing capability of the industry can be further improved.

## Methods

### Training team building

**Fig. 2:**
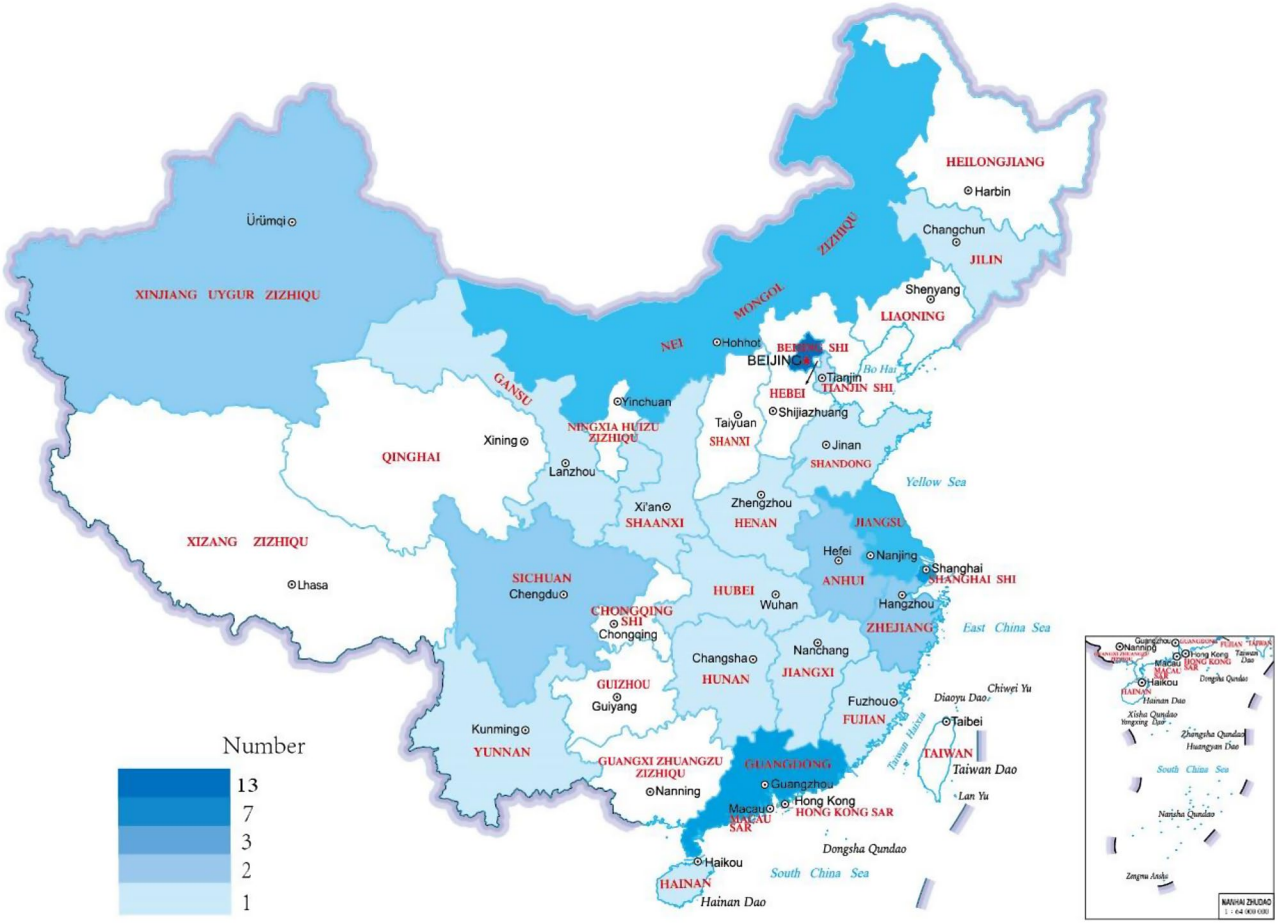
Coverage area distribution of expert teams. The areas with colors were covered by the teaching team of the project. Data Credits: All the city administrative boundaries are based on the standard map from the service website of Ministry of Natural Resources of the People’s Republic of China which number is GS (2019) 1676

The training program of experimental diagnostics open course is led by Peking Union Medical College Hospital and sponsored by the China Primary Health Care Foundation. The experimental protocols, training plans, and educational methodologies delineated herein have undergone thorough review and have received formal approval from the China Primary Health Care Foundation. And we had the informed consent of all the participants. The core teaching expert team was selected from hospitals and institutions across the country with mNGS localization platforms and some industry clout. The first group of experts is mainly from 46 hospitals and institutions, including Peking Union Medical College Hospital, West China Hospital of Sichuan University, Zhongda Hospital of Southeast University, Zhongshan Hospital of Fudan University, The Second Clinical College of Guangzhou University of Chinese Medicine, Wuhan Center for Clinical Laboratory, The First Affiliated Hospital of USTC, etc. which covers 21 provinces and municipalities (Fig. 2), including 53 core experts and 28 young backbone members. Of the core specialists, 43 are in clinical laboratories, 4 are in infection departments, 3 are in precision medicine, 2 are in central clinical laboratory departments and 1 is in pathology. The main trainee-participants are technical personnel of clinical laboratories in the surrounding teaching hospitals, clinical personnel and other relevant personnel who need to take part in the training. The actual participants are recommended by the surrounding hospitals of each teaching hospital to participate in the training, and each hospital recommends at least one participant. The questionnaire survey is completed before the formal training, including the professional background of the trainees in each hospital and their understanding of the practical application of mNGS localization, so as better to develop the training content. In June 2021, in order to better carry out training activities, 10 experts from 7 hospitals who undertake training courses in 2021 visited MGI Customer Experience Center (Wuhan) to personally practice the training process and put forward suggestions for improvement of the training process, providing reliable support for the smooth implementation of the project.

### Training platform

We aim to set up an online platform micro medical (https://www.microonline.cn/hdyy.html), explore pathogenic microorganisms in the diagnosis of frontier technology progress, converge infectious disease diagnosis and treatment quality learning resources and related subjects. All students and practitioners interested in learning more about mNGS are welcome to visit the site.

The offline platforms for the first batch of training courses were provided by West China Hospital of Sichuan University, Zhongda Hospital of Southeast University, Zhongshan Hospital of Fudan University, The Second Clinical College of Guangzhou University of Chinese Medicine, Wuhan Center for Clinical Laboratory and The First Affiliated Hospital of USTC, which were assisted by BGI PathoGenesis Pharmaceutical Technology, BGI-Shenzhen.

### Training course development

To ensure uniform and standardized training content, the project uses unified training curriculum and video, and a core team of experts prepares and standardizes the course content. More than 40 domestic experts participated in an online preparatory meeting in May 2021, where the syllabus and training methods were discussed. Most experts agree that lecture topics should be categorized, and suggest adding lessons such as sharing clinical practice cases. They also claimed teaching hospitals needed to additionally familiarize themselves with the content of the teaching. Finally, we discussed the origin of the instability of the bacterial reservoir in the context of the localization of the mNGS technique. We determined the final training content after three rounds of discussion and revision of the curriculum content. According to their respective research directions, experts from the laboratories, clinical, research and development, scientific research and joint laboratories of the Chinese universities determined the tasks of courseware fabrication and theoretical teaching courseware fabrication, respectively. Through two rounds of collective lesson preparation and courseware modification, this project has produced a course resource chain with “localization construction and application of mNGS” as the main line, providing three-dimensional and granular course resources for the implementation of training classes, and aiming to create a professional and accurate learning home for the majority of clinical microbiology and anti-infection workers.

As technology keeps updating and iterating, online courses are in a constant state of improvement. As of August 30, 2022, 22 courses had been uploaded, covering basic concepts, sampling, laboratory construction, wet and dry experiments, clinical applications, scientific research design, guideline consensus, etc. (Table 1). Ensuring that students and practitioners who interested in learning mNGS can learn systematic theory quickly and conveniently.

**Table 1:** List of online training courses

Number	Course Title
1	Basic concepts of mNGS detection
2	Interpretation of expert consensus on clinical application of mNGS in detecting infectious pathogens at China
3	mNGS localization platform application experience sharing
4	Introduction to the knowledge of mNGS related microbiology
5	Research design of mNGS in the field of infection
6	Introduction of PMseq sequencing platform and technology principle
7	Human microecology and establishment of mNGS background bacterial bank
8	Clinical application of mNGS
9	mNGS Specimen Collection (Medical Care Edition)
10	Pretreatment of specific sample types in mNGS experiments
11	mNGS laboratory site and personnel requirements
12	MGISP-100 Automation platform application guide
13	Precautions for library pooling scheme
14	Double barcode cyclization technology
15	High human background specimen and technical treatment
16	Quality control and Common problems solving in the process of wet experiment
17	Analysis principle and application instructions of PMseq bioinformatics analysis software
18	This section describes related parameters in the data disconnection report
19	Bioinformatics analysis of data from sequencer
20	Identification of mNGS species based on homologous fuzzy comparison
21	Bioinformatics analysis of emerging pathogen
22	Demonstration of PMseq wet experiment operation

The offline training is guided by the localization construction and application of mNGS, and the relevant training curriculum content is designed. The main purpose of this course is to help trainees understand the mNGS technology, facilitate local clinical construction and application of mNGS, and thus improve clinical diagnosis capabilities. To deepen the trainees’ understanding of the practical application process of the mNGS technique, the training is divided into two parts: theoretical explanation and on-site practical operation. With the trainees as the main body, the hospital was guided to construct a 3-day/period offline classroom training course. The specific course content is given in Table 2. To match the actual situation, the content of the course may be increased or decreased, while the main content is retained.

**Table 2:** Content of offline training courses

Training time	Training content	
Day 1 Sample preprocessing combined with nucleic acid extraction	Theoretical explanation	Conceptual design of pathogen mNGS laboratory; Sample quality control before testing: sampling points and sample quality requirements; PMseq detection principle and key points of operation; Operational video learning;
	Field operation	Sample preparation: key points of sample pretreatment and nucleic acid purification
Day 2 Library construction combined with computer introduction	Field operation	Library construction and DNB preparation: Operational essentials of field library construction; Computer sequencing and report interpretation exercise: report interpretation exercise;
Day 3 Report interpretation, operation video comments	Q&A discussion	Report interpretation exercise: disembark data report interpretation exercise
	Assess	Assess
	Operation video comment	Video playback: student operation video comments
	Award certificate	Award certificate

### Training pattern

We adopt models that combine online and offline classes, build diverse learning platforms and approaches, and strive to create small-scale and efficient training class criteria. Combined with the online platform, small-scale offline training courses have been held in numerous places across the country, with 10 to 12 sessions held each year. Each session will be conducted by a teaching hospital as the primary training unit, and the training scale will be approximately 8–10 people per session. Students accumulate theoretical knowledge through online theoretical learning followed by participated in small-scale training sessions with designated units of the program and conducted theoretical and practical evaluations.

### Training assess

All students are required to take online courses and master basic theoretical reserves before offline classes begin. Survey statistics of student completion were also obtained through questionnaires. At the end of the offline class, there are practical test results and theoretical test results (paper form). The whole offline classroom practical operation was recorded, and the performance of the students was evaluated and reviewed based on the results of the data analysis of the student operations, which were taken as the actual evaluation results. After training, theoretical examinations are conducted through written examinations. Based on the content of the clinical construction and application of mNGS localization, a total of 55 questions were set, consisting of 20 multiple-choice questions, 20 fill-in-the-blank questions, 10 judgment questions, and 5 short-answer questions. The examination papers were collected and counted by each teaching hospital. More than 80 points will be eligible and certificates will be issued to eligible students.

### Training implementation evaluation

Online classrooms: statistics of online course page views and overall page views; The questionnaire was used to investigate student satisfaction with the course content and the use of the platform.

Offline classrooms: statistics of overall student attendance and training assessment pass rate; Statistics of the growth rate and usage variation of localization platforms; The feedback of students on the training course is understood by means of a questionnaire and individual soliciting of opinions. The questionnaires will be handed out after the training and will be completed and submitted by the trainees independently. The solicitations for individual opinions were chosen at random to understand the actual feelings of the trainees, training difficulties and opinions.

## Results

### Survey the situation before training

Before each training session, a questionnaire was conducted to investigate the professional background of the hospital students and their understanding of the practical applications of mNGS localization. A total of 59 questionnaires were collected in this survey. The results showed that the top 3 courses that students wanted to gain most were pathogen metagenomic sequencing laboratory design scheme, mNGS wet experiment (DNA&RNA) process, and sequencing data interpretation/reporting practice. In addition, we learned that most of the students had microbial-related work experience and PCR certificates. In the online examination before training, 44.19% (19/43) failed to pass the theoretical examination.

### Overall training class development

In June 2021, the China Primary Health Care Foundation, together with Peking Union Medical College Hospital and additional hospitals, launched a training program for the China High Throughput Sequencing and Diagnostic Capacity Improvement Program for Infections. It is planned to carry out 10–12 offline trainings every year for a period of 5 years starting from 2021, aiming to improve the detection and diagnosis level of teaching hospitals, student hospitals and the industry, create industry training benchmarks, establish industry training standards, promote the clinical application of mNGS technology and advance contemporary technologies, and promote the development of the industry.

**Fig. 3:**
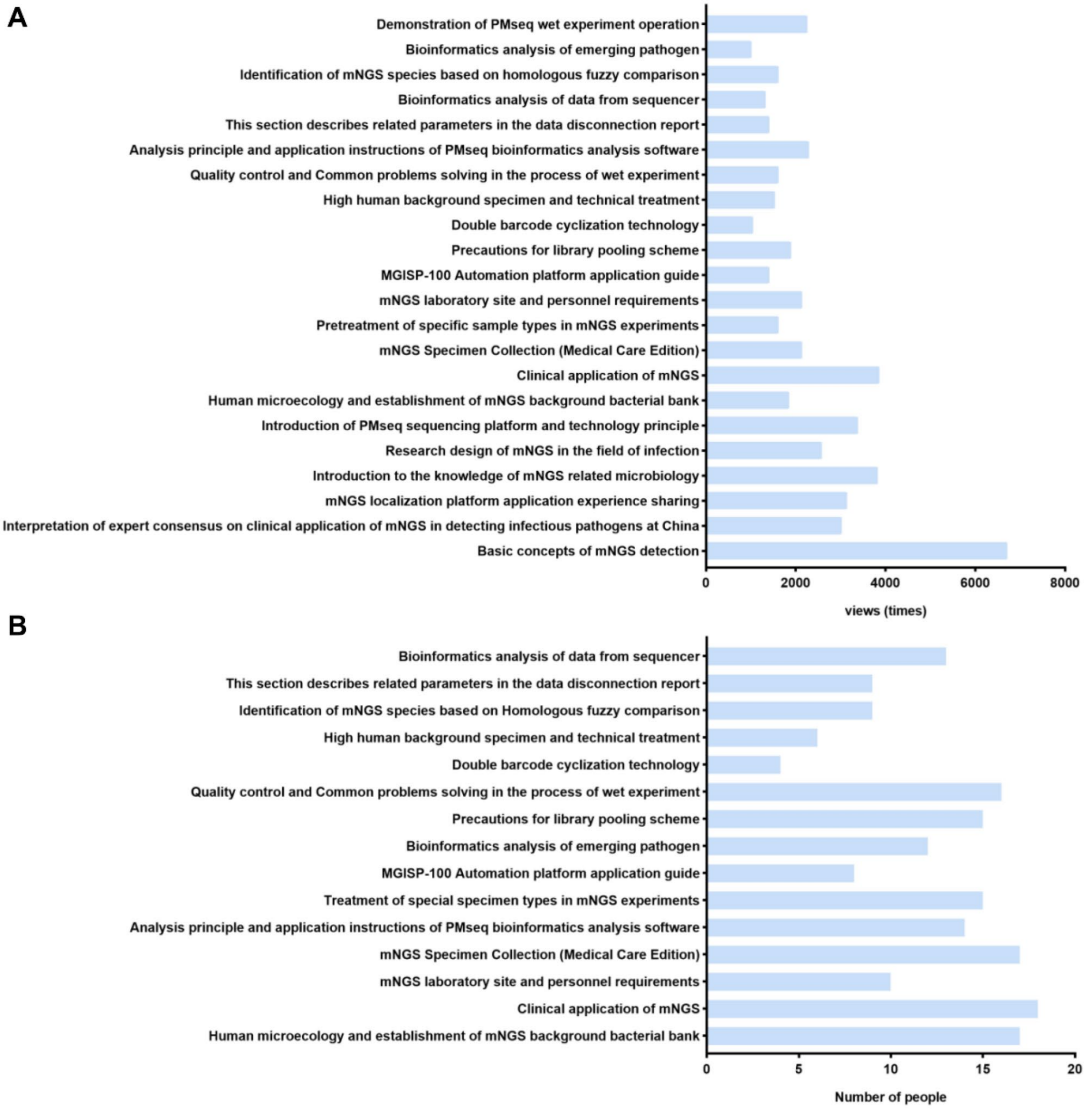
Satisfaction survey of online training class. (**A**) Statistics of online course Page Views (January 1-August 30, 2022); (**B**) Online courses that help students the most

The total page views of online classes have reached 51,500 times from January 1to August 30, 2022, and the average daily page views are about 214 times. The top 3 courses in page views (Fig. 3A) are Basic concepts of mNGS detection (6707 times), Clinical application of mNGS (3856 times), and Introduction to the knowledge of mNGS related microbiology (3815 times). In the offline questionnaire, 55 students answered questions about the online course. 65.45% claimed to have taken an online course before offline training. The top 3 online courses selected by students with the most help is Clinical application of mNGS, mNGS Specimen Collection (Medical Care Edition), Human microecology and establishment of mNGS background bacterial bank (Fig. 3B).

By August 30, 2022, a total of six small-scale offline training sessions had been held, each lasting three to four days and involving 8 to 14 participants. Finally, about 67 trainees who had completed both online and offline courses from 67 hospitals in different provinces have participated in the training with the passing rate of the trainees is 100% (Table 3). In addition to the prescribed theoretical and practical parts, the training content of each course is combined with the real-world situations and characteristics of the teaching hospitals to add some course content, such as progress in the practical application of mNGS in clinical practice.

**Table 3:** Overall training participation

Number	Training course (teaching hospital)	Participating hospitals (Number)	Participants (Number)
1	West China Hospital, Sichuan University	9	9
2	Zhongshan Hospital, Fudan University	12	12
3	Zhongda Hospital, Southeast University	12	12
4	The Second Clinical College of Guangzhou University of Chinese Medicine	8	8
5	Wuhan Center for Clinical Laboratory	14	14
6	The First Affiliated Hospital of USTC	12	12
Total		67	67

**Fig. 4:**
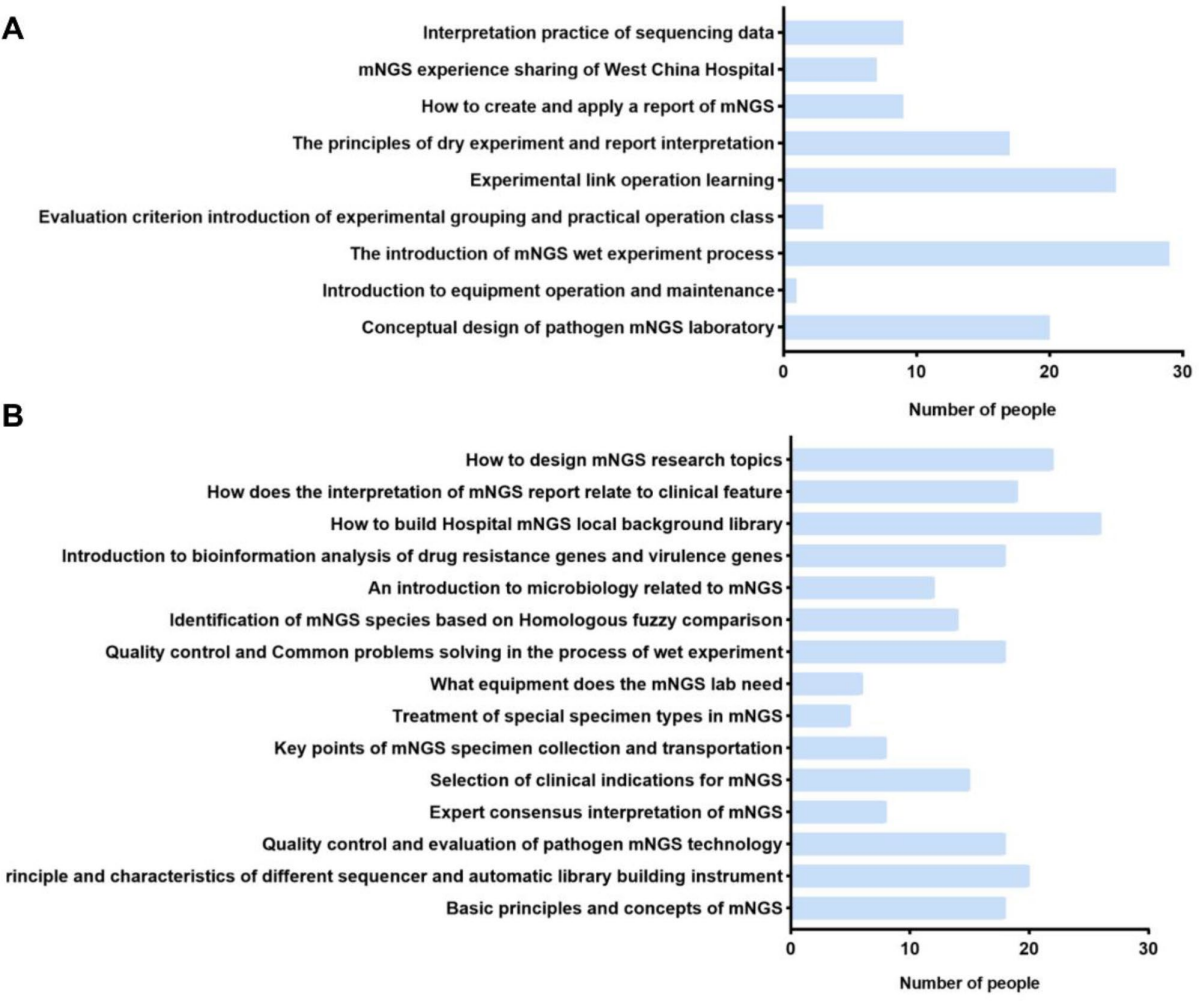
Satisfaction survey of offline training class. (**A**) Offline course satisfaction; (**B**) Expected course content

In the questionnaire survey results after training (Fig. 4A), the top 3 courses in terms of satisfaction with offline courses were the introduction of mNGS wet experiment process, Experimental link operation learning, and Conceptual design of pathogen mNGS laboratory. The top 3 courses expected to be added (Fig. 4B) are How to build Hospital mNGS local background library, How to design mNGS research topics, Principle and characteristics of different sequencer and automatic library building instrument.

### Evaluation of training effectiveness

In pre-training assessment and evaluation of some students, the qualification rate was only 55.81%, but after training, it reached 100%. Only 17 of the 67 student hospitals participated in the training have mNGS localization platforms before training, but none of them were operational. After the training course, until August 30, 2022, the localization platform had been put into use in 1 student hospital, 2 hospitals had added the mNGS localization platform for admissions, while 3hospitals have expressed strong intention to do that. The six training sessions had a wide-ranging impact followed by 20 reports of Self-Media, 33 of Mass-Media, 3 of Video Channel, 5 of Vertical -Media and 53 of portal, news and health media etc.

A total of 51 questionnaires were collected after training. Most trainees indicated that they were extremely satisfied with the content of the training course and felt that they had gained a lot. Some students also suggested that the schedule was too tight. Training objectives should be extended to include additional clinical staff as required.

To the training content should be added the explanation of the report, the technical principles of the sequencer, and additional contents.

### Training in experience and innovation

#### Brainstorm ideas to ensure the effectiveness and accuracy of training

Online preparatory meetings are held to gather wisdom and eventually form a fixed training pattern and training content; Training sessions are held to identify possible problems in the training process in advance and improve them to ensure a smooth progress of the offline training sessions; Meetings will be held to enhance the impact of training courses and expand their reach. A pre-training study is conducted to understand the professional background of the training subjects, understand the content related to the localization application of mNGS, and provide accurate teaching to improve the effectiveness of the training.

#### Assist in the construction of grassroots inspection staff

The training was given to relevant laboratory or clinical staff of the mNGS laboratory of the hospital, especially primary level laboratory staff with high seniority. Grass-roots inspectors, who had been working on the front lines for a long time, had fewer opportunities for all kinds of business training, and were hesitant to promote the use of improved techniques, methods, and instruments. The training established an online platform to provide students with a modern learning platform and additional learning resources. Offline centralized training streamlines the training cycle and increases the feasibility of grass-roots surveyors to participate in training. Strengthening the professional training of grassroots inspection personnel can effectively drive the overall improvement of the professional level of the department.

#### Standardize training content to improve course quality

Based on the training context and training objectives, the overall training content is determined, and a core team of experts in the field develop courseware and instructional plans, standardize course content, and improve course quality. Combined with the actual clinical situation and the results of questionnaire survey, we added the course content that the students are concerned about or interested in which is closer to the reality and more intuitive, and improved the depth and breadth of training.

#### Multi-training mode to enhance the interest and effectiveness of trainees during training

In order to enhance the efficiency and impact of the training, a combination of online and offline training modes is adopted, in conjunction with the current era of epidemic normalization and multimedia. Online classes cover a wide range of course resources and are convenient for students not limited to time and place to watch and learn, improving the learning environment and increasing learning efficiency. Face-to-face interaction with teachers in offline classes gives students a sense of engagement. The hands-on operation allows students to intuitively experience the mNGS and gain a deeper understanding. The training method combines theory with practice to improve student engagement.

#### Small-scale training courses should be held in sub-centers to improve organizational efficiency and coverage.

It is difficult to carry out the training uniformly, considering that the whole country is too wide. Small-scale training courses are mainly conducted by teaching hospitals at regional centers. The training is carried out in multiple centers across the country, with an impact covering 21 provinces and autonomous regions across the country. A reasonable training scale can provide a facilitating teaching and learning environment for both teachers and students. Small-scale training courses which effectively reduce organizational and administrative difficulties are more conducive to improving student participation and improving student-teacher interaction.

#### Accurate training evaluation provides effective support for continuous improvement of the training system

Before the training, a questionnaire was conducted to understand the professional background of the trainees, their understanding of the training content and interest in the course content. Precise planning of training content, timely adjustment of training direction and improvement of training experience of trainees. After the training, the opinions and suggestions of the trainees are collected to understand the real thoughts of the participants during the training sessions. The training schedule is improved based on relevant content, and the training system is continuously upgraded.

## Discussion

Since the national implementation of the training program based on the mNGS localization construction and application construction, a total of six training courses have been conducted until August 30, 2022, covering multiple hospitals and institutions in multiple provinces and cities, and was well received in many ways. The cadets achieved a 100% attendance and pass rate; Some student hospitals have started to build and use the mNGS localization platform. For example, the localization platform of the Sichuan Provincial People’s Hospital has been put into operation, and the second People’s Hospital of Chengdu and the affiliated hospital of the North Sichuan Medical College have added the localization platform for admissions. Industry attention and influence is also gradually increasing, and more hospital institutions are liking to participate in the training. As the training experience continues to accumulate, the training model and the training curriculum system also improve. In addition to increasing the standardization and completeness of training content, the 6th at Anhui Provincial Hospital was integrated with an online platform to realize the first live broadcast of the training. At present, the domestic training on the application of mNGS technology in clinical practice is mainly focused on the theoretical level, but this training program adopts the way of theory and practice to make the trainees understand the principles and application scenarios of mNGS technology more intuitively, which can help the trainees better apply mNGS technology in clinical. This project training can also more effectively realize the application value of localization of mNGS detection technology, help the accurate diagnosis and treatment of infectious diseases in various departments and effectively improve the diagnostic capacity of infectious diseases in hospitals, so as to treat diseases in a timely and effective manner, reduce the cost of patient treatment, and improve the quality of life. Continued training courses have enabled more hospitals to perform mNGS technical diagnostics, further improving the level of detection and diagnosis in the industry and effectively supporting the standardization of training in the industry.

Of course, there are still many shortcomings in this program, such as the lack of assessment mechanisms in online courses, the need to improve teaching content and the lack of multiple and effective supervision and incentive mechanisms. This is also a direction that future training programs need to further explore and improve. Therefore, in the future, it is necessary to continuously strengthen training, keep content updated, further improve standardization construction. Second, according to the national requirements for the application of a continuing education program, the location of the program, the overall requirements, the content requirements and the courseware requirements are in good agreement with each other. ​It has the basics to apply for national continuing education programs and has great potential for future development. Finally, a crucial prerequisite for precision medicine is accurate diagnosis. It has become a trend in medical laboratories to use modern technologies and methods to provide accurate information for clinical use. In addition, the development of Laboratory Developed Test (LDT) has adapted to the development of precision medicine tests to some extent. Therefore, the construction of mNGS localization platforms must be an essential part of future precision medicine detection, and it is also worth focusing on how to promote the use of mNGS localization platforms across the country in the future.

## Conclusion

The online classroom combined with the offline small-scale training mode mentioned in this study well adapts to the new situation of the post-epidemic era and the multimedia digital era, solves the training difficulties in the current industry, and effectively promotes the localization construction and application of mNGS in hospitals. This is the first mNGS training program to be systematically standardized worldwide. It has great potential for future development and deserves further promotion.

## Data Availability

The datasets used and/or analysed during the current study are available from the corresponding author on reasonable request.

## Abbreviations

mNGS: Metagenomic next-generation sequencing
LDT: Laboratory Developed Test
